# High sensitivity rate-integrating hemispherical resonator gyroscope with dead area compensation for damping asymmetry

**DOI:** 10.1038/s41598-020-80885-y

**Published:** 2021-01-26

**Authors:** Wanliang Zhao, Hao Yang, Fucheng Liu, Yan Su, Chong Li

**Affiliations:** 1 Nanjing University of Science and Technology, Nanjing, 210094 China; 2 Shanghai Aerospace Control Technology Institute, Shanghai, 201200 China; 3 Ocean University of China, Qingdao, 266100 China

**Keywords:** Aerospace engineering, Electrical and electronic engineering, Mechanical engineering

## Abstract

The rate-integrating gyroscope (RIG) operation is considered as the next generation architecture for hemispherical resonator gyroscopes (HRGs) with advantages of direct angle measurement and unlimited dynamic range. However, this RIG operation requires high symmetry for the HRG device and the damping mismatch of the two gyroscopic modes will result in a dead area problem. This work analyzes the error mechanism of the damping asymmetry induced dead area and proposed a novel virtual procession compensation method for HRG RIG. The simulation proves the existence of the dead area as the theory predicted. More importantly, the experimental HRG RIG platform with the proposed compensation method can significantly expand the dynamic range with accurate angle measurement and overcome the problem of dead area. The earth rotation is accurate measured which is the first time that captured by a RIG scheme as a state-of-the-art result.

## Introduction

The hemispherical resonator gyro (HRG) is type of precision inertial sensor that has the advantages of high reliability, long life time cycle and low bias drift performance^1^. It is widely utilized in aerospace applications, defense technology and advanced industry^2,3^. It has a merit of low bias instability (BI) of below $0.001^\circ$/h that enables the inertial unit measuring the attitude of the carrier accurately with a navigation level performance^4,5^. Though the HRG shows its admirable BI performance, the advanced applications are expecting it can break through its bottle neck of limited dynamic range. It’s well known that the HRG is widely used in measuring the attitude of satellites, not only the advantages that discussed above, but the more important reason is that satellites’ relatively low dynamics fits the measurable region of HRGs. It’s highly expecting that HRGs can be adopted to more critical applications with their low BI and high reliability, but the limited dynamic range is the barrier must be overcame.

The conventional way to drive the HRG is named as rate mode. The primary vibration mode is excited at its resonant frequency and the leaves the sense mode to detect the rotation rate. Under this architecture, the characteristics of highly symmetry and ultra high quality factor become a double edge sword. On one hand it effectively improves the signal sensitivity to reach a very low angle rand walk, but on the other hand, it’s sacrificing the dynamic range and bandwidth^6^. The force-to-rebalance technique used to be a effectively expand the dynamic range^7^. It’s using the principle of closed-loop system shaping that applying a control force to maintain the sense mode stationary^8^. Although FTR has proved its effectiveness and reliability, its total enhancement to the dynamic range is limited by the stability issue of the closed-loop system.

The rate-integrating gyroscopic (RIG) or namely whole-angle (WA) operation is a novel solution for HRGs to significantly expand the measurable dynamic range and bandwidth^9^. Unlike the rate mode, the RIG method is using the free response of the gyro system that considers the two vibration modes equally^10^. It’s observing the precession angle in the gyro to determine the external rotation angle to provide angle measurement with unlimited range. This architecture requires highly symmetry of the device to prevent error occurs^11^. This principle can be applied to all kinds of Coriolis vibratory gyros (CVG), includes micro-electro-mechanical system (MEMS) gyros^12^. Since MEMS gyros tend to be low-cost and batch manufactured, its precision and symmetry are worse to HRG to be a better candidate for RIG^13,14^. Nevertheless, MEMS gyro and HRG are sharing the same fundamental principle, so the HRG RIG gains knowledge to improve its performance, etc. error analysis^15,16^, energy control^17,18^, bias compensation^19^, advanced circuit design^20^, mode decoupling^21,22^ and quadrature cancellation^23^.

The dead area problem has became increasingly important to HRG RIG, because HRG aims for high-end applications, which is not reported and investigated yet. More specifically, the sensor output is not responding to the small changes of angle at certain areas. This measurement inaccuracy will amplify the attitude/positioning error in the HRG cooperated navigation system.

This study analyzes the principle of the dead area in HRG RIG , proposed a novel self-calibrated method and induces the state-of-the-art HRG RIG performance. The detailed contributions of this manuscript are summarized as follows:

1. The working principle and error mechanism of HRG RIG is comprehensively presented and analyzed. In particular, the influence of the damping mismatch and its induced error with dead area problem is analyzed in depth.
2. A novel, robust and practical architecture with energy control, quadrature elimination and virtual precession compensation is proposed and implemented for high performance HRG RIG operation. This operation and calibration architecture can effectively sustain the HRG in the RIG mode and solve the asymmetry errors.
3. Experimentally verified the enhancement of the HRG RIG with a state-of-the-art measurement sensitivity and dynamic range expansion. The dead area problem is solved by the virtual rotation method with experimental verification and it’s the first time that the earth rotation is detected by the RIG type of gyroscopes.

The remainder of this manuscript gives the detailed theoretical analysis, verification and discussions.

## Dynamics analysis of HRG RIG operation

### Ideal dynamics of symmetry HRG

**Figure 1:**
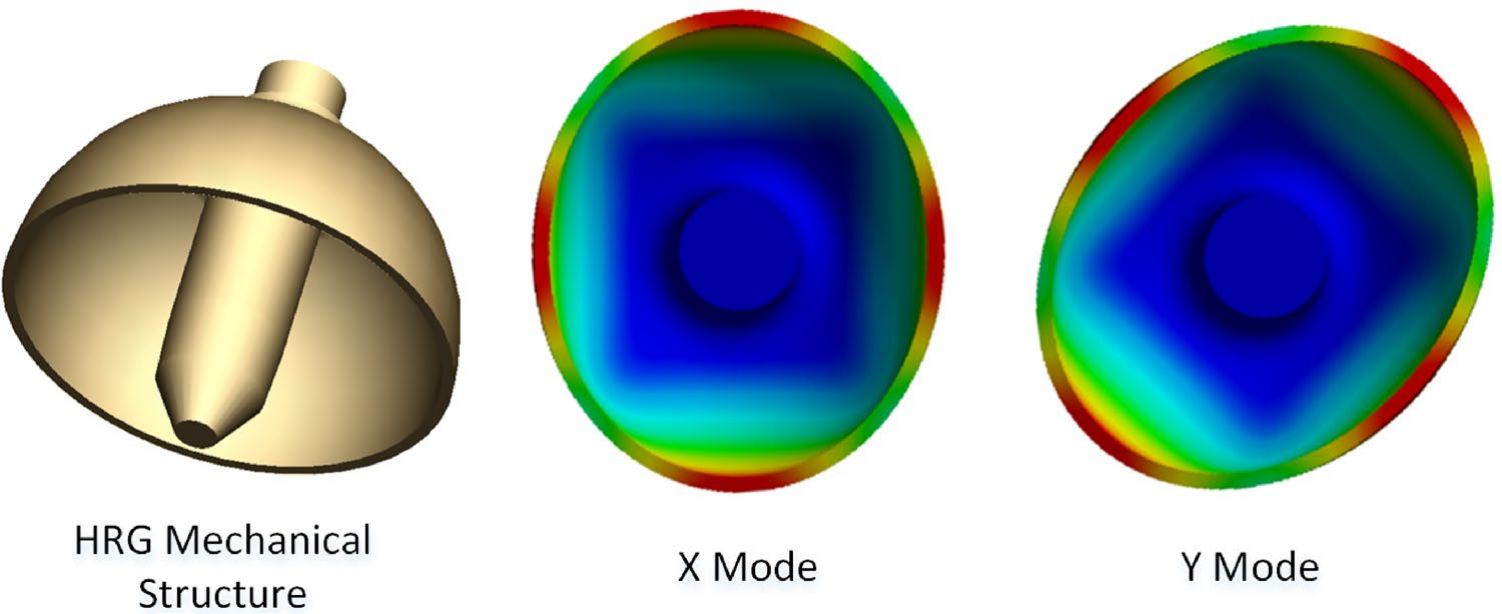
Degenerated gyroscopic modes of the HRG.

The mechanical structures and its two degenerated gyroscopic modes are shown in Fig. 1, which is typically made by fused-silica or metal^24,25^. The HRG has a series of advantages include low energy dissipation rate, large signal pickup electrodes and high symmetry to make it an ideal candidate for precision resonant gyroscope. The $n=2$ vibration mode is chosen for the gyroscopic operation.

**Figure 2:**
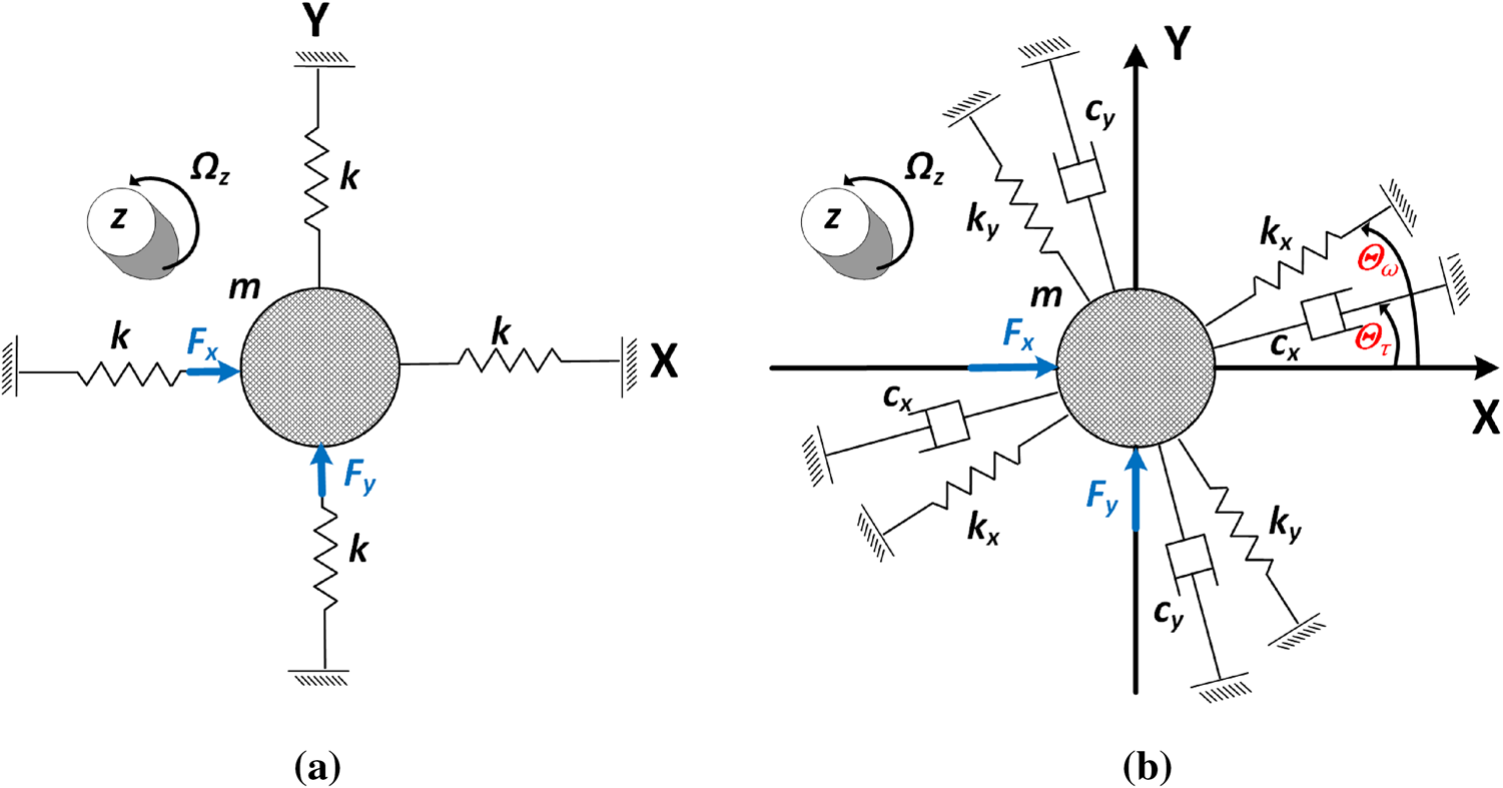
Comparison of (**a**) ideal HRG model and (**b**) HRG model with asymmetry components.

The equivalent vibration model can be established in Fig. 2a as an ideal symmetric system with 2-degrees-of-freedom (DOF). The differential equations that describes the :

1 $$\begin{aligned} \begin{aligned}{}&m\ddot{x}+kx=F_x-2m\lambda \Omega _z{{\dot{y}}}\\&m\ddot{y}+ky=F_y+2m\lambda \Omega _z{{\dot{x}}}, \end{aligned} \end{aligned}$$

where *x* and *y* are the generalized coordinates the two modes (*X* and *Y*) of the HRG, *m* is the proof mass, *k* is the stiffness and are equal in the two modes. $F_x$ and $F_y$ are the artificial forces that applied to the HRG, $\lambda$ is the angular gain that determined by the vibration mode of the mechanical structure design. Specifically, for $n=2$ mode that used in the HRG, $\lambda =0.275$ would be the case. $\Omega _z$ is the rotation that the HRG needs to be measured in the *Z* axis. The damping terms and cross coupling terms are assumed to be ignored in this ideal situation.

The resonant frequencies of *X* and *Y* are the same that described by:

2 $$\begin{aligned} \begin{aligned}{}&\omega =\sqrt{k/m}. \end{aligned} \end{aligned}$$

where $\omega$ is the resonant frequency.

Unlike a conventional HRG that utilizes the forced response of the 2-DOF vibration system, the RIG mode is essentially using the free response of (1) without direct interactions of $F_x$ and $F_y$. The initial conditions *x*(0), *y*(0) must not be zeros to let the free vibration happens. The normalized free response solution of (1) can be obtained by the method of undetermined coefficients:

3 $$\begin{aligned} \begin{aligned}{}&x(t)= \alpha \cos\left( \theta _0+\int \lambda \Omega _z dt\right) \cos ( \omega t)\\&y(t)= \alpha \sin\left( \theta _0+\int \lambda \Omega _z dt\right) \cos ( \omega t), \end{aligned} \end{aligned}$$

where $\alpha$ is the vibration amplitude that depends on the initial condition and $\alpha \cos(\theta _0+\int \Omega _z)$ and $\alpha \sin(\theta _0+\int \Omega _z)$ needs to be further processed. Considering the fact that the dynamics of $\omega$ is much faster than $\Omega _z$, so they can be divided into ”fast” and ”slow” variables. The slow variables can be extracted by using in-phase/quadrature (IQ) demodulation method and then calculating the angle:

4 $$\begin{aligned} \begin{aligned}{}&\theta _0+\int \lambda \Omega _z dt=arc \tan\left( \frac{\alpha \cos(\theta _0+\int \lambda \Omega _z dt)}{\alpha \sin(\theta _0+\int \lambda \Omega _z dt)}\right) . \end{aligned} \end{aligned}$$

**Figure 3:**
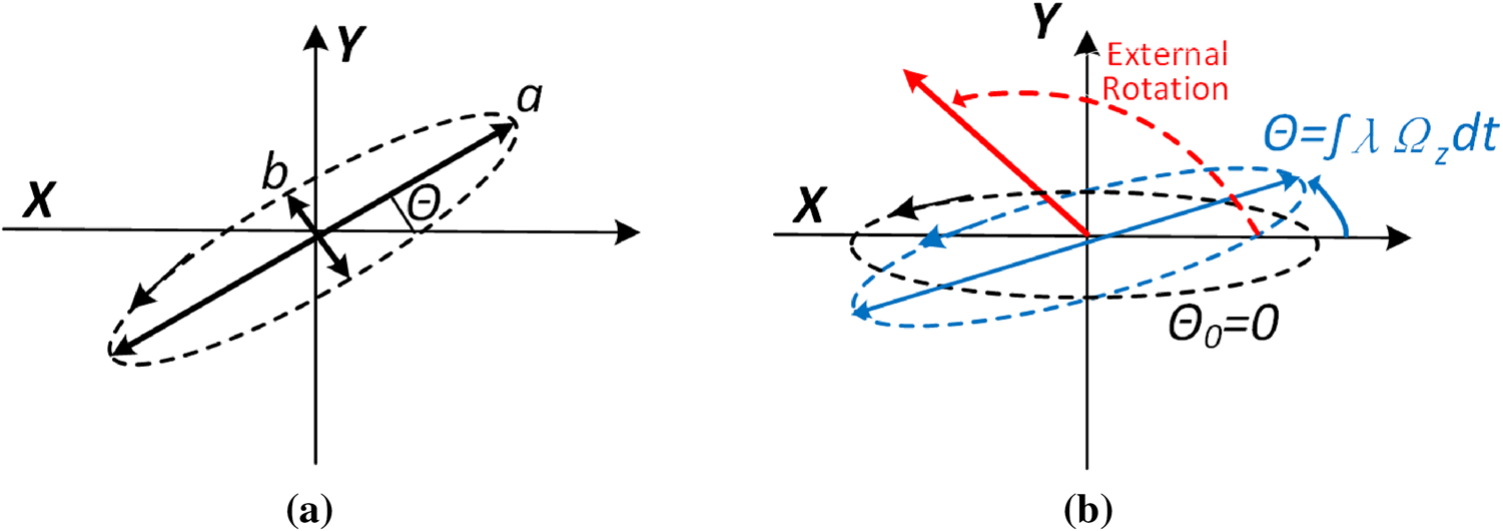
Mass center orbit of the HRG RIG.

The relationship between the precession angle and the external physical rotation rate can be described by:

5 $$\begin{aligned} \begin{aligned}{}&\theta =\int \lambda \Omega _z dt, \end{aligned} \end{aligned}$$

where $\theta$ is the precession angle. and is demonstrated in Fig. 3. $\theta$ reflects to the angle rotation in the Z axis instead of rate and can be calculated by measuring the amplitude *x*(*t*) and *y*(*t*) according to (4). It’s feasible to extract $\left\| x(t) \right\|$ and $\left\| y(t) \right\|$ through in-phase quadrature (IQ) modulation process.

The principle of RIG can be illustrated in Fig. 3. Normally, the mass center of the HRG is doing a periodical elliptical motion with a initial precession angle of $\theta _0$ and *a*, *b* are the major and minor axes. Figure 3b shows the process of angle precession. Assuming that the HRG is initially at the orbit angle of $\theta _0=0$ and an external rotation of total $145^\circ$ is applied to the HRG RIG. The orientation of the vibration wave is following this external rotation but with a ratio of 0.275 due to the inherent characteristics of the $n=2$ mode.

### HRG with asymmetry error factors

However, this promising RIG architecture for HRG can not be implemented in a straight forward way because the existence of the asymmetry errors which can be described by:

6 $$\begin{aligned} \begin{aligned}{}&m\ddot{x}+c_x{{\dot{x}}}+k_xx+c_{yx}{{\dot{y}}}+k_{yx}y=F_x-2m\lambda \Omega _z{{\dot{y}}}\\&m\ddot{y}+c_{y}{{\dot{y}}}+k_yy+c_{xy}{{\dot{x}}}+k_{xy}x=F_y+2m\lambda \Omega _z{{\dot{x}}}, \end{aligned} \end{aligned}$$

where the spring stiffness $k_x, k_y$ and damping terms are not balanced to each other in real practice because the imperfection of the materials and fabrication process. Thus, the resonant frequencies of the two modes are no longer matched:

7 $$\begin{aligned} \begin{aligned}{}&\omega _x=\sqrt{k_x/m}\\&\omega _y=\sqrt{k_y/m}.\\ \end{aligned} \end{aligned}$$

$k_{xy}$ = $k_{yx}$ and $c_{xy}=c_{yx}$ are the stiffness and damping coupling terms that caused by the azimuth mismatch as shown in Fig. 2b. The damping and stiffness azimuths are divided from the principal axis of the vibration with angles of $\theta _\tau$ and $\theta _\omega$ to induce the coupling terms. The HRG system equation can be better described by^11^:

8 $$\begin{aligned} \begin{aligned}{}&\ddot{x}+\left( \frac{1}{\tau _1}+\frac{1}{\tau _2}\right) {{\dot{x}}} +\left( \frac{1}{\tau _1}-\frac{1}{\tau _2}\right) ({{\dot{x}}}\cos(4\theta _\tau )+({{\dot{y}}}\sin(4\theta _\tau )) +\left( \frac{\omega _x^2+\omega _y^2}{2}\right) x\\&\quad -\frac{\omega _x^2+\omega _y^2}{2}(x\cos(4\theta _\omega )+y\sin(4\theta _\omega )) =\frac{F_x}{m}-2\lambda \Omega _z{{\dot{y}}} \\&\ddot{y}+\left( \frac{1}{\tau _1}+\frac{1}{\tau _2}\right) {{\dot{y}}} -\left( \frac{1}{\tau _1}-\frac{1}{\tau _2}\right) (-{{\dot{x}}}\sin(4\theta _\tau )+({{\dot{y}}}\cos(4\theta _\tau )) +\left( \frac{\omega _x^2+\omega _y^2}{2}\right) y\\&\quad -\frac{\omega _x^2+\omega _y^2}{2}(-x\sin(4\theta _\omega )+y \cos(4\theta _\omega )) =\frac{F_y}{m}+2\lambda \Omega _z{{\dot{x}}}, \end{aligned} \end{aligned}$$

where $\tau _1$ and $\tau _2$ are the decaying time constants fo *x* and *y*. Another set of definitions are used to better analyze the system:

9 $$\begin{aligned} \begin{aligned} \frac{1}{\Delta \tau }&=\frac{1}{\tau _1}-\frac{1}{\tau _2} \\ \epsilon &= \frac{1}{2\Delta \tau }, \end{aligned} \end{aligned}$$

where $\epsilon$ is a new definition for the later analysis.

Observing the established dynamics model, an intuition would be that these asymmetry errors has significantly changed the dynamics of the HRG RIG and will lead the measurement errors.

## Asymmetry error analysis and calibration techniques

### Damping asymmetry induced threshold rotation and dead zone problem

Because the existence of the damping mismatch terms, the precession angle dynamics is described by

10 $$\begin{aligned} \begin{aligned}{}&{{\dot{\theta }}}=-\lambda \Omega _z-\frac{1}{\Delta \tau }\sin(4\theta -4\theta _{\tau }). \end{aligned} \end{aligned}$$

The solution of (10) will depend on the external input conditions.

1. When $\left| \lambda \Omega _z \right| < \frac{1}{\lambda \Delta \tau }$, it can be solved as^26^:
  11 $$\begin{aligned} \begin{aligned}{}&\theta =\theta _{\tau }+ \frac{1}{2}a\tan\left( \sqrt{\left( \frac{\epsilon }{\lambda \Omega _z}\right) ^2-1} +\frac{\epsilon }{\lambda \Omega _z} -\frac{2\sqrt{\left( \frac{\epsilon }{\lambda \Omega _z}\right) ^2-1}}{1+c_0e^{ 2\lambda \Omega _z 2\sqrt{\left( \frac{\epsilon }{\lambda \Omega _z}\right) ^2-1}t } }\right) , \end{aligned} \end{aligned}$$
  where $c_0$ is a coefficient that described by:
  12 $$\begin{aligned} \begin{aligned}{}&c_0=ln\frac{ \sqrt{\left( \frac{\epsilon }{\lambda \Omega _z}\right) ^2-1} +\left( \tan2\theta _0-\frac{\epsilon }{\lambda \Omega _z}\right) }{ \sqrt{\left( \frac{\epsilon }{\lambda \Omega _z}\right) ^2-1} -\left( \tan2\theta _0-\frac{\epsilon }{\lambda \Omega _z}\right) } \end{aligned} \end{aligned}$$
  As $t \rightarrow \infty$, the stead state of (11) would be:
  13 $$\begin{aligned} \begin{aligned}{}&\theta _{\infty }=\theta _\tau -arc\sin\left( \frac{\lambda \Omega _z}{\epsilon }\right) . \end{aligned} \end{aligned}$$
  Because $\lambda \Omega _z$ is small and the 2nd term in (13) can be approximated as:
  14 $$\begin{aligned} \begin{aligned}{}&\theta _{\infty } \approx \theta _\tau , \end{aligned} \end{aligned}$$
  which indicates that the HRG RIG is not responding to the small rotation rates and a dead area of $\pm \frac{1}{\lambda \Delta \tau }$ is conducted.
2. When $\left| \lambda \Omega _z \right| > \frac{1}{\lambda \Delta \tau }$, the solution of the original one described in (10) would be complex. However, its characteristics can be analyzed by the following equation which is obtained through a symbolic computation tool:
  15 $$\begin{aligned} \begin{aligned} \tan(2\theta )&= 2\sqrt{1-\left( \frac{\epsilon }{\lambda \Omega _z}\right) ^2} \quad \tan\left( \quad -2\lambda \Omega _z t \sqrt{1-\left( \frac{\epsilon }{\lambda \Omega _z}\right) ^2} \right. \\&\quad \left. +a\tan\frac{\tan(2\theta _0-2\theta _\tau )-\frac{\epsilon }{\lambda \Omega _z}}{\sqrt{1-\left( \frac{\epsilon }{\lambda \Omega _z}\right) ^2}} \quad \right) +\frac{\epsilon }{\lambda \Omega _z}, \end{aligned} \end{aligned}$$
  which indicates that when $\left| \Omega _z \right|>> \frac{\epsilon }{\lambda }$, (15) can be further simplified as:
  16 $$\begin{aligned} \begin{aligned}{}&\tan(2\theta )=\tan(-2\lambda \Omega _z t + a\tan(2\theta _0)).\\ \end{aligned} \end{aligned}$$
  The dynamics of the $\theta$ become ideal with a larger input rate $\Omega _z$ and its dynamic cam be described by:
  17 $$\begin{aligned} \begin{aligned}{}&{{\dot{\theta }}}\approx -\lambda \Omega _z+\frac{\epsilon ^2}{2\lambda \Omega _z}.\\ \end{aligned} \end{aligned}$$
  Both (15) and (17) indicate that when the input rate is sufficiently large, the non-ideal terms will be cancelled and the HRG RIG is close to the ideal behavior.

The influence of the existence of damping mismatch can be summarized that it will result in the dead area problem to limit its performance toward high-end applications, which desires an effective solution.

### Dead-zone calibration with virtual precession

According to the analysis in (eqref), the HRG is blind to small angle rotations due to the damping miss match conditions. To overcome this problem, a virtual precession solution is proposed and its basic formula is given as below:

18 $$\begin{aligned} \begin{aligned}{}&\theta =\theta _0+\int \lambda \Omega _z dt + \int \lambda \Omega _v dt, \end{aligned} \end{aligned}$$

where $\Omega _v$ is the virtual rotation that generated from the electrical system of the HRG. Thus, the physical angle $\int \lambda \Omega _z dt$ then is superposing with this virtual rotation to prevent the break the threshold of the undesired dead-zone. The physical angle can be recovered by subtracting the known virtual rotation from the observed precession angle.

Considering feasibility in the engineering practice, the virtual precession rate can be designed as a constant that $\Omega _v=\Omega _{vir}$ and (18) can be reorganized as:

19 $$\begin{aligned} \begin{aligned}{}&\theta =\int \lambda (\Omega _z + \Omega _{vir}) dt. \end{aligned} \end{aligned}$$

The electrical Coriolis force that actually generating this virtual precession can be expressed as:

20 $$\begin{aligned} \begin{aligned}{}&f_{xv}=A_e \Omega _{vir} {{\dot{y}}}\\&f_{yv}=-A_e \Omega _{vir} {{\dot{x}}}, \end{aligned} \end{aligned}$$

where $f_{xv}$ and $f_{yv}$ are part of $f_x$ and $f_y$ as well as other control signals, and $A_e$ is the electrical to force coefficient that depends on the actual circuit and HRG mechanical properties.

### HRG RIG operation architecture with dead area compensation

**Figure 4:**
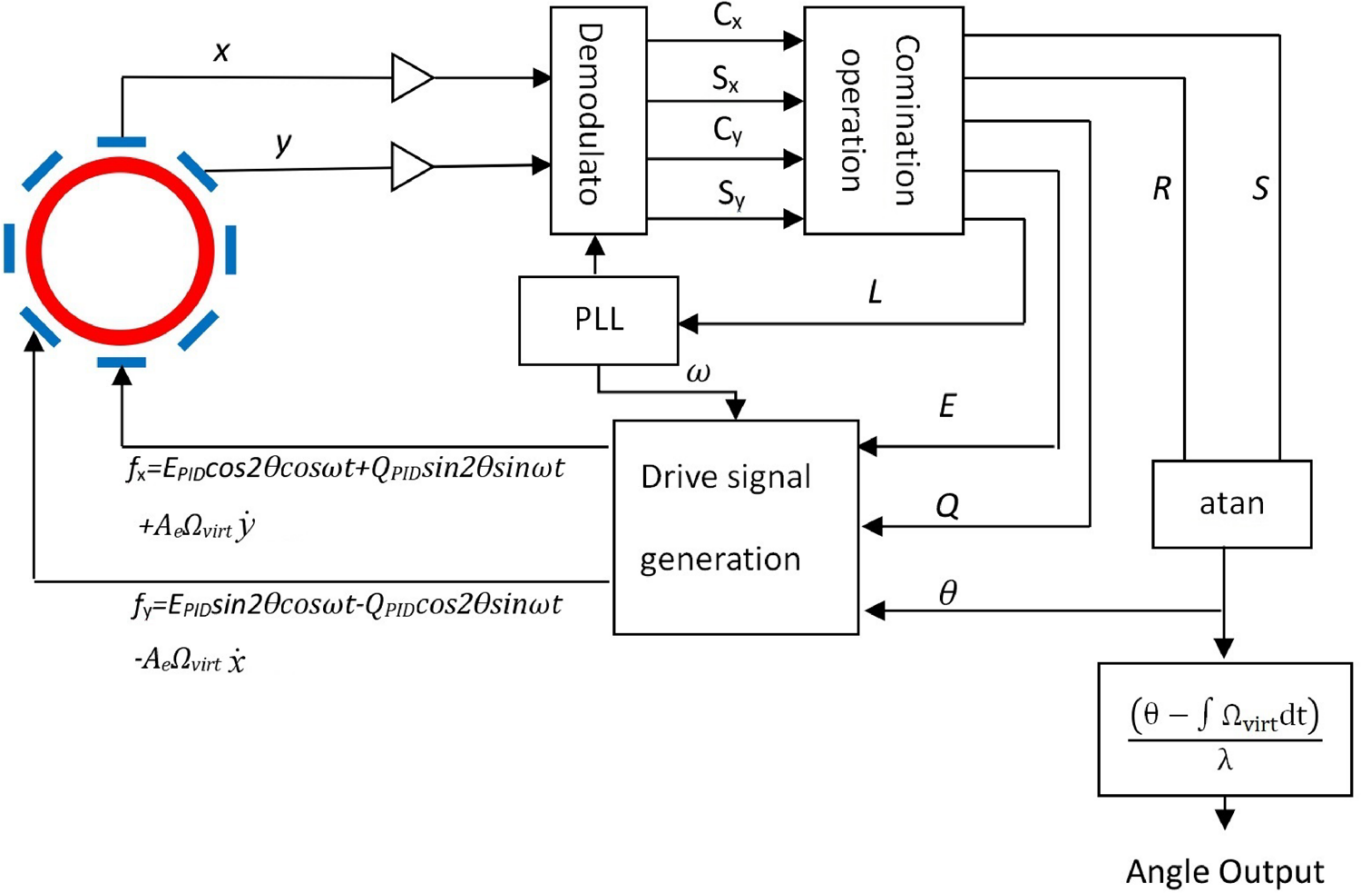
Interface architecture for the HRG RIG.

To implement the dead area calibration technique along with other important control loops, a FPGA based digital system is developed which is shown in Fig. 4.

The output signal *x* and *y* are amplified through the front-end electronics for the further signal processing blocks. The demodulation is based on the in-phase quadrature (IQ) principle, so $C_x$, $S_x$, $C_y$ and $C_s$ are in in-phase and quadrature components of the two gyroscopic modes. To extract the desired information to support the RIG operation, the following terms are calculated as:

21 $$\begin{aligned} \begin{aligned}{}&E=C_x^2+S_x^2+C_y^2+S_y^2\\&Q=C_xS_y-C_yS_x\\&R=C_x^2+S_x^2-C_y^2-S_y^2\\&S=2(C_xC_y-S_xS_y)\\&L=2(C_xS_x-C_yS_y),\\ \end{aligned} \end{aligned}$$

where *E* is the total kinetic energy of the HRG, *Q* is the quadrature motion, *L* is used for the drive loop^23^, *R* and *S* are the parameters that used to calculate the precession angle $\theta$ by using the following formula:

22 $$\begin{aligned} \begin{aligned}{}&\theta =\frac{1}{4} a\tan\left( \frac{S}{R}\right) .\\ \end{aligned} \end{aligned}$$

The energy *E* is the fundamental control loop for the RIG operation that it must maintains as a constant level to neither degraded to the forced response mode nor the motion decays to zero. More specifically, the energy control is a landmark between rate gyro and rate-integrating gyro because this loop sustains the free vibration of the HRG without a timing limitation. On the other hand, continuously pumping energy without control will result in a rate type gyro. The calculated *E* will go through a PID controller with a set-point $E_{PID}$ and then redistributed to the two modes:

23 $$\begin{aligned} \begin{aligned}{}&f_{xe}=E_{PID}\cos(2\theta )\cos(\omega t)\\&f_{ye}=E_{PID}\sin(2\theta )\cos(\omega t), \end{aligned} \end{aligned}$$

where $E_{PID}$ is the control effort to sustain the free vibration of the HRG RIG.

The quadrature motion of the gyro needs also to be eliminated by generating a control input $Q_P{PID}$ and allocate this effort to X/Y modes:

24 $$\begin{aligned} \begin{aligned}{}&f_{xq}=Q_{PID}\sin(2\theta )\sin(\omega t)\\&f_{yq}=Q_{PID}\cos(2\theta )\sin(\omega t), \end{aligned} \end{aligned}$$

where $Q_{PID}$ is the quadrature cancellation component of the drive signal.

Since the virtual precession is already discussed, so the overall control effort in the based band will be eventually synthesized based on (20), (23) and (24):

25 $$\begin{aligned} \begin{aligned}{}&f_{x}=f_{xe}+f_{xq}+f_{xv}=E_{PID}\cos(2\theta )\cos(\omega t)+Q_{PID}\sin(2\theta )\sin(\omega t)+A_e \Omega _{vir}{{\dot{y}}}\\&f_{y}=f_{ye}+f_{yq}+f_{yv}=E_{PID}\sin(2\theta )\cos(\omega t)+Q_{PID}\cos(2\theta )\sin(\omega t)-A_e \Omega _{vir}{{\dot{x}}}. \end{aligned} \end{aligned}$$

*L* is the indicator for the resonant frequency tracking and it’s a phase lock loop (PLL) type implementation in this design. The two modes of the HRG can be fine tuned through the electrostatic spring softening effect that $\omega = \omega _x \approx \omega _y$^27^ . The generated digital sinusoidal signal is also used to up convert the control signals in (25).

The most important information from the measurement system would be the precession angle measurement which is described in (22). It’s notable that the measured angle is mixed with the virtual precession angle and the desired physical angle should be computed as:

26 $$\begin{aligned} \begin{aligned}{}&\theta _p=\frac{1}{\lambda }\left( \frac{1}{4} a\tan\left( \frac{S}{R}\right) -\int \Omega _{vir} dt\right) ,\\ \end{aligned} \end{aligned}$$

where $\theta _p$ is the real physical angle that needs to be measured through the HRG RIG.

## Validation

### Simulational verification

A set of simulation studies were performed in advance of the experimental validation, because the simulation can provide more ideal configuration than the real device. In the real practice, there’s no perfect symmetry HRG devices and so the theoretical differences between conventional rate and RIG architectures are difficult to be observed. The dead area problem is also expected to be simulated in this ideal environment before the experimental phase to improve the productivity. The software environment was MATLAB 2019b and the gyro model was built in the MATLAB SIMULINK toolbox. To assure the reliability of the simulation study, the simulated HRG had a mode-matched resonant frequency of 5 KHz, nominal Q of 8,000,000, which are close to the typical HRG devices.

**Figure 5:**
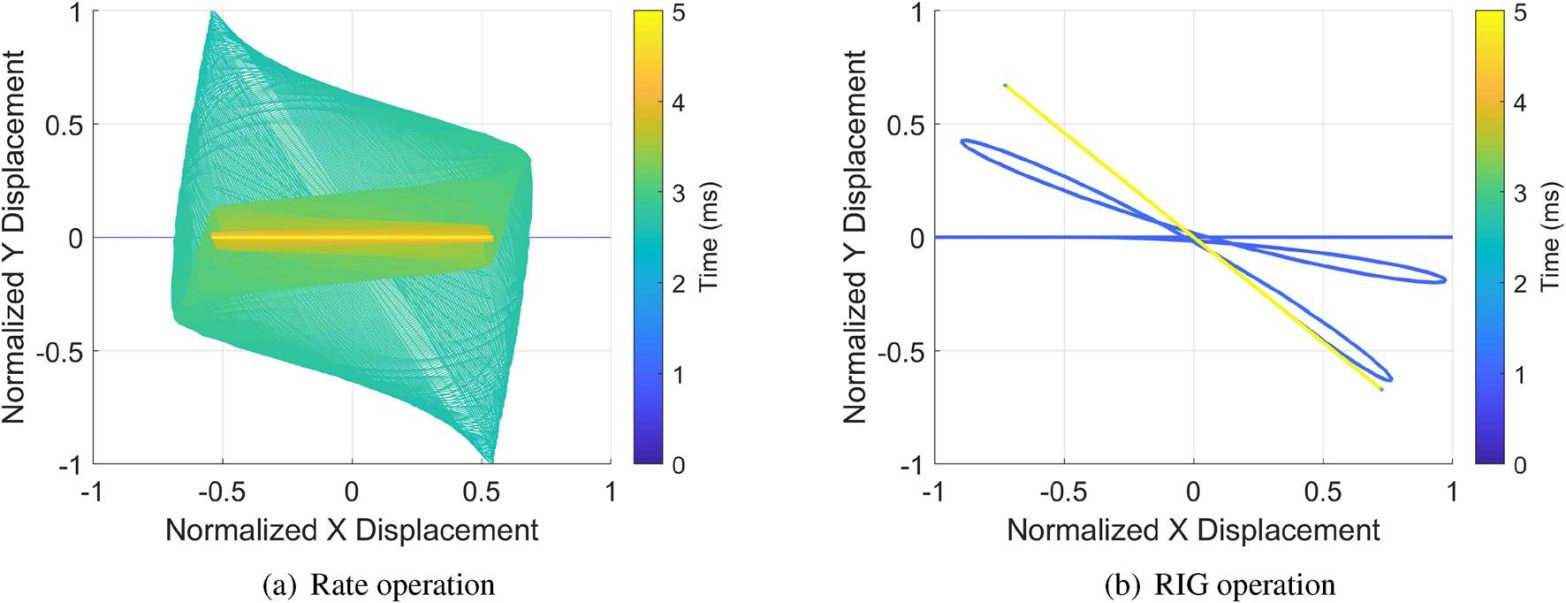
Comparison of the orbits between rate and RIG mode under a step input of $45^\circ$.

The simulation initiated with a perfect symmetry gyro to demonstrate the idea of HRG RIG operation which is shown in Fig. 5. A step input of $180^\circ$ rotation was applied to the rate/RIG modes of HRG. The results show that the response in the sense mode had very limited motion as small signals to hurt the signal to noise ratio and acted as a rate gyro. The orbit angle of RIG mode was responding to the rotation angle as a direct angle measurement sensor. This comparison also indicates that the RIG architecture also has potential advantage of higher signal to noise ratio. The conventional rate architecture divide the gyro into the drive mode and sense mode, and continues pump energy into the primary derive mode while the motion of rate signal modulated sense mode is very small. This relatively weak signal leads to inherent difficulties to the electronics design to reduce the noise. On the contrary, the RIG architecture treats the two modes equally and the external input is determined by examining the total energy distribution on the two modes, so the signal to noise ratio can be maximized.

**Figure 6:**
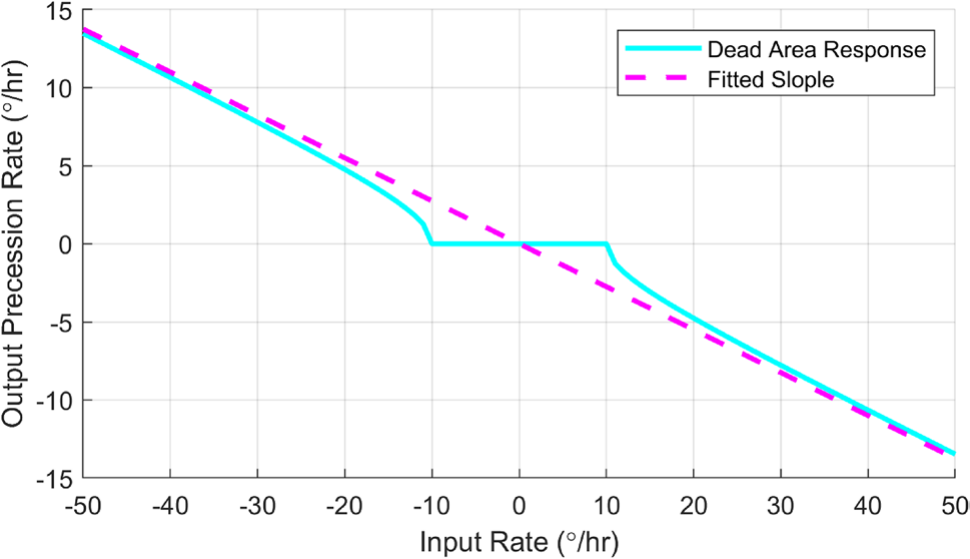
The dead-zone effect of HRG with damping mismatch.

Then a Q mismatch was introduced to the simulation with a induced dead area threshold of $10^\circ$/h. The deviation of damping axis and the principal axis, $\theta _\tau$, equals $30^\circ$. The input rotation rate-gyro precession rate output relationship is shown in Fig. 6. The dash line was the ideal gyro input-output function with a slope of 0.275 since it was using the $n=2$ mode. The solid line was generated by solving the differential equation (10) numerically. It’s obvious that the overall scale factor between input and output was linear, but it was not respond to the small rotation as the theory predicted.

**Figure 7:**
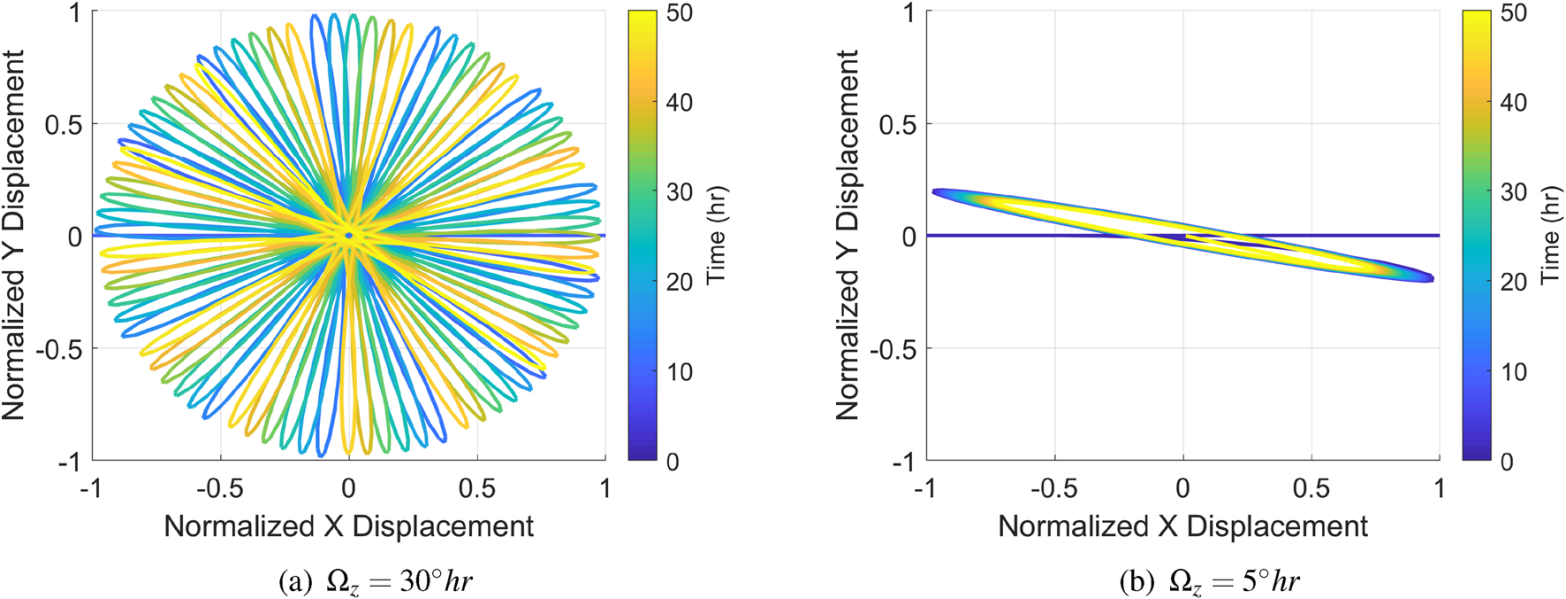
Dead area simulation results with input rates larger and smaller than the threshold.

**Figure 8:**
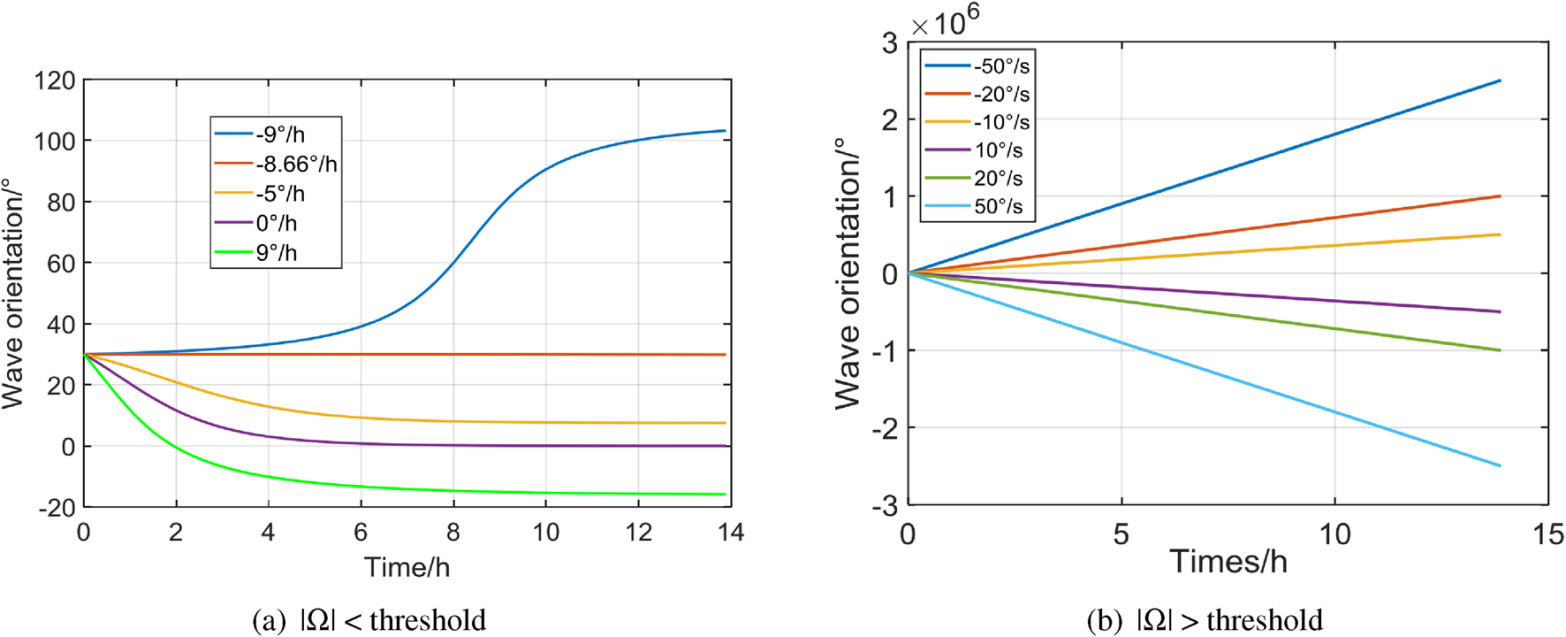
Time domain responses of HRG with input behind/beyond the dead-zone threshold.

To better illustrate the existed problem, a set of time domain comparison simulation trajectories was collected and is shown in Figs. 7 and 8. Constant rotation rates of $\Omega _z=30^\circ$/h and $\Omega _z=5^\circ$/h were applied to the HRG RIG, which were inside/outside of the dead area, where the results are shown in Fig. 7. Even with the damping asymmetry, the precession angle of the HRG RIG followed the $\Omega _z=30^\circ$ rotation input normally because this rate was outside of the dead area. However, the orbit angle barely responded to the $\Omega _z=5^\circ$ with very limited angle precession since the the motion of the wave was trapped by the dead area.

To comprehensive discover the problem of dead area, another set of constant rotations were applied to the gyro and the initial precession angle was same as $\theta _\tau = 30^\circ$ for the convenience as shown in Fig. 8. When the input rate was less than the threshold, the gyro precession angle responded deadly slow. Even the maximum rate, $9^\circ$ was nearly reaching the threshold, the total precession angle was no more than $70^\circ$ after a 14 h operation. On the contrary, when the rotation was in the outside the threshold, the output trajectory worked as an almost ideal gyro and the slope fitted the input very well. The higher rotation rates up to $\pm \,50^\circ /\text {h}$ also proved the desired linearity.

Based on the simulation studies, it can be concluded that the dead area problem do exist in the damping mismatched HRGs. The input rate that behind/beyond the threshold will result in a big difference in the HRG measurements and leaving this unsolved will let HRG RIG stay out of the candidate in the high precision navigation applications. The further calibration solution and its results is given in the later subsection.

### Experimental validation

**Figure 9:**
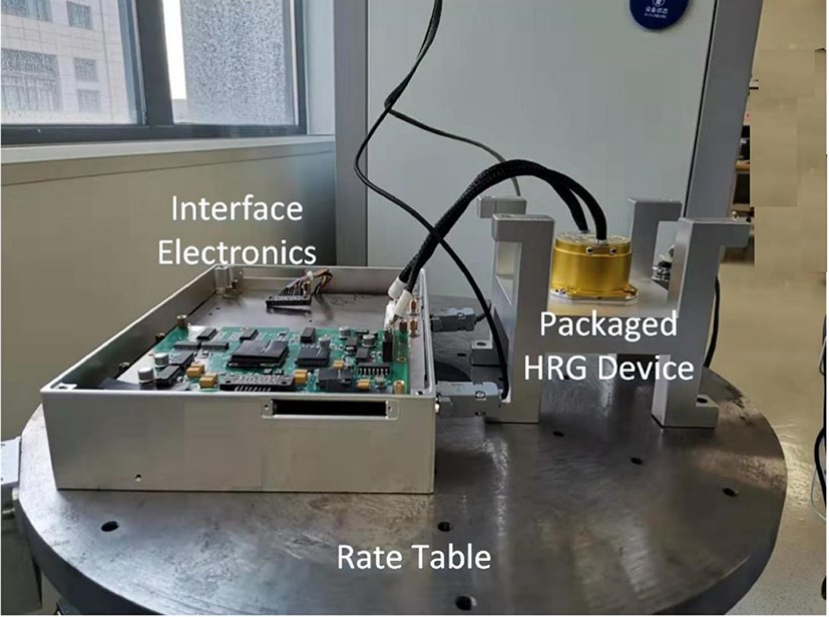
Experimental setup of the HRG RIG.

A customized industrial level HRG platform was developed to evaluated the proposed solution regarding to the dead area problem to conduct a high performance HRG RIG system. The experimental setup is shown in Fig. 9. The HRG device was well packaged and sealed to adopt to the environmental variation and prevent the air damping to reduce the Q. The interface electronics had a core of Xilinx FPGA with high speed digital processing capability to implement the proposed architecture and the precession rate table can provide the accurate rotation input. The generated drive voltage signals were amplified through the power electronics and conducted the electrostatic forces that can simulate the resonator effectively. The motion of the device can be detected by monitoring the signal pick-up electrodes. The HRG device was sealed in the vacuum to retain the low energy dissipation rate. The device and the interface system were mounted on the rate table so variety of physical rotation can be applied to this sensor.

**Figure 10:**
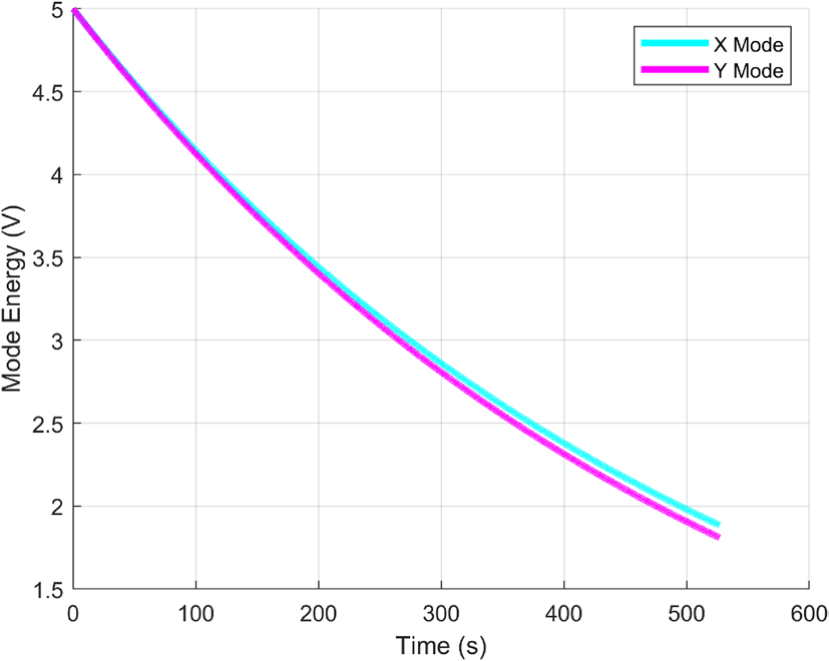
Ring down measurement of the two degenerated modes of the HRG.

The basic characterization of this HRG was performed to optimize the performance in the later experiments. The resonant frequency of the HRG was 4.5 kHz and the mode split of X and Y was less than 0.001 Hz after an electrostatic tuning through a frequency sweep type measurement. Because the HRG had very large Qs for the two modes that reached million level, the frequency sweep test can no longer applied because the high Q induced resonant peaks were too sharp to be accurately captured with limited data points. Thus, a ring down measurement was applied to get the accurate Qs for the two modes as shown in Fig. 10. Each of X/Y mode was initially excited at its resonant frequency by using the PLL and then released the control amplitude to zero to let the totally energy decay. Then the Q of the mode can be calculated by observing the energy decay time. The measured decay times of X and Y mode were 522s and 520s that represented $Q_x =7{,}800{,}000$ and $Q_y =7{,}340{,}000$. These characterizations demonstrated that the HRG was in a well tuned condition and well balanced except for the Q mismatch.

**Figure 11:**
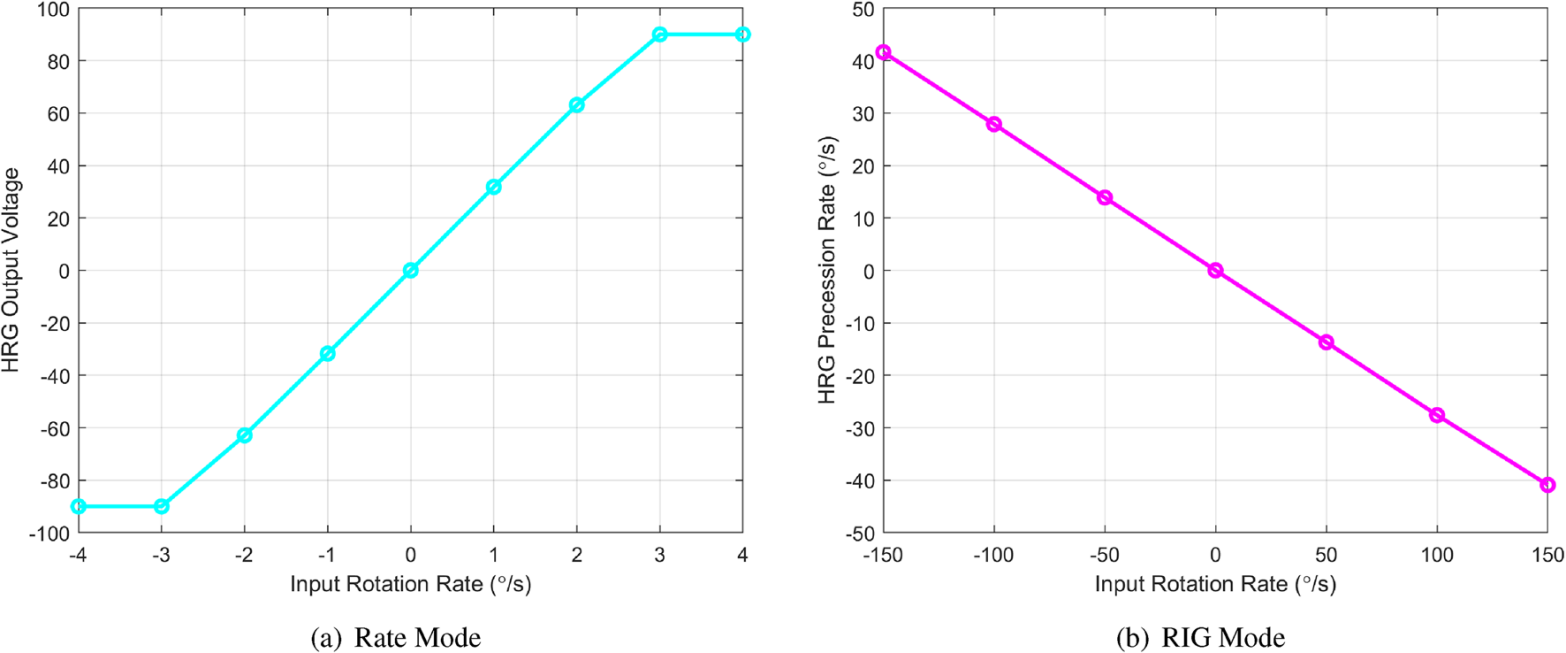
Dynamic range comparison of the conventional rate mode and RIG of the HRG.

As claimed that the RIG architecture can significantly expand the dynamic range of HRG, a comparison was done between the RIG and conventional rate mode as shown in Fig. 11. The rotation rates were provided by the precision rate table. In the rate mode, the output signal of the HRG had became saturated when the rotation rate was greater than $3^\circ /\text {s}$, because it was under the mode match operation principle and the 8 million level high Q will greatly reduce the dynamic measurement range. On the contrary, the RIG provided a linear dynamic range of $150^\circ /\text {s}$, which is a remarkable 50x improvement. This improvement was resulted from the inherent advantage of the RIG architecture that the external rotation will be determined by direct measuring the precession angle. The nonlinearity of the scale factor of the HRG RIG is less than 0.1% according to the fit result. This significant improvement can broaden the market of the HRG from limited aerospace to other advanced industrial or defense applications with high rotation dynamics.

**Figure 12:**
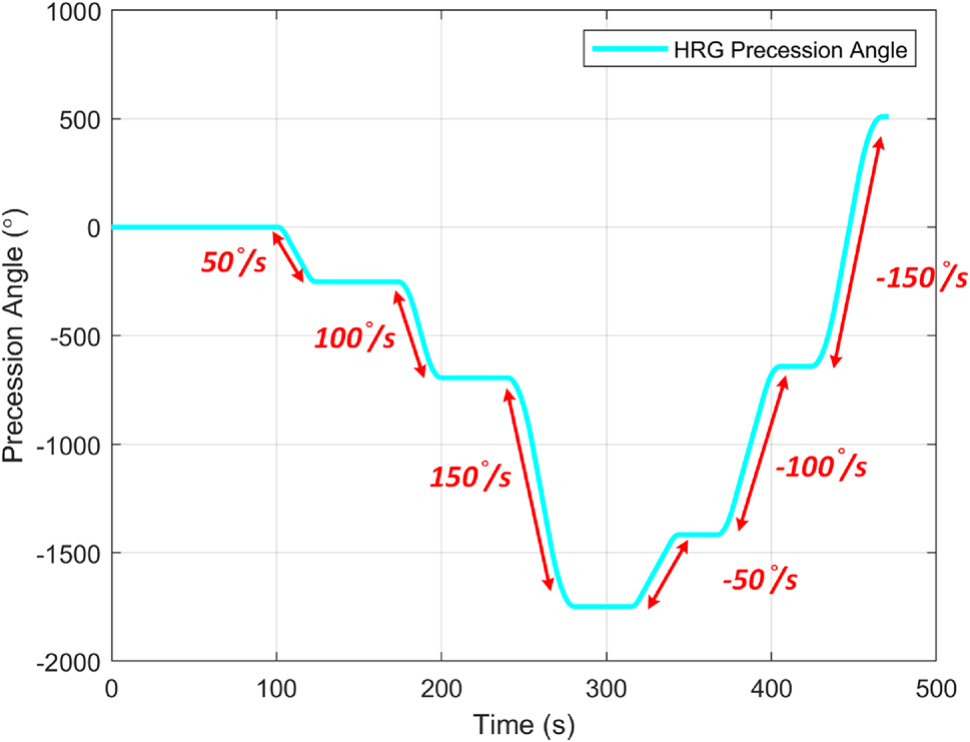
Step responses of the HRG RIG with different rotations.

Another “step response” rotation test was proceeded to verify the bias stability of the HRG with the proposed interface architecture, which is shown in Fig. 12. The rate table applied different rates ($\pm 50 ^\circ/\text{s}$, $\pm 100 ^\circ/\text{s}$ and $\pm 150 ^\circ/\text{s}$,) as steps to the gyro and stayed steady for about 50s for each step. For MEMS based RIG operations, it’s very hard to retain the output level with a constant angle but will drift to the principle axis with lower damping ratio instead. For the case with the proposed architecture with HRG, the angle output maintained its level without any obvious drifting when the rate table stopped. It fully proved the functionality and effectiveness of the architecture and it’s the first research showing the stable steady state performance for a HRG.

**Figure 13:**
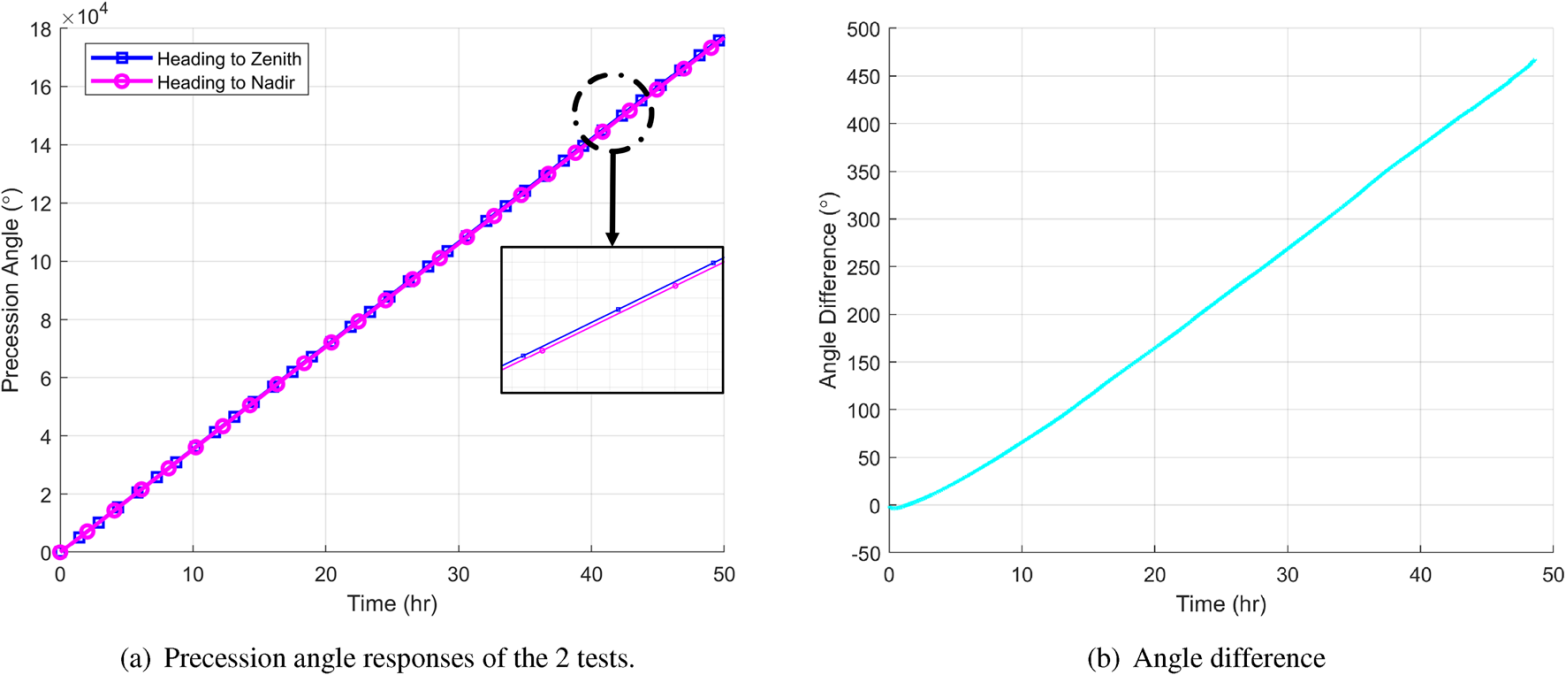
Dead area compensation verification experiment by using the earth rotation.

The dead area compensation problem was verified through measuring the earth rotation. It’s well known that the earth is rotating with a rate of $7.5^\circ /\text {h}$ as a small rate, which is inside the dead area of the HRG RIG. The experiment was utilizing this small earth rotation as the arbitrary input for a flip-over comparison. The HRG z-axis was pointing to the zenith in this first round for 50 h and then heading to the nadirz which is a up-side-down situation, where the two rounds were running with the virtual rotation rate of $1860^\circ /\text {h}$. So the output of 1st measurement was the virtual rotation plus the earth rotation and the 2nd one would be the virtual rate minus the earth rotation. If the two measurements were doing the difference, a difference of $2\times 7.5 ^\circ /\text{h} \times 0.275 = 4.125 ^\circ /\text{h}$ should be obtained. Fig. 13 demonstrates these two tests that by doing the difference, a rotation rate of $4.2^\circ /\text {h}$ was captured by the differential type of comparison. It successfully proved that the dead area problem is solved by the proposed architecture and it’s the first time that CVG RIG has been able to measure the earth rotation as a state-of-the-art result. In contrast, the difference of these two configurations presented a zero output when the virtual rotation was turned off, which further proved the principle of the damping asymmetry induced dead area error and the effectiveness of the proposed method.

## Conclusion

The operation principle and error mechanism of HRG RIG with damping/Q asymmetry and the compensation method is comprehensively investigated in this work. When the input physical rotation is smaller than the Q mismatch induced dead area threshold, the HRG RIG is deadly respond to it. Thus, a virtual electrical precession can be applied to break through this barrier to provide precise angle measurement. The experiments proves that the HRG RIG with this solution can significantly expand the measurement dynamic range and captured the earth rotation as a state-of-the-art result. The suggested future work would be further explore and optimize the error mechanisms of HRG RIG and this work can be adopted to MEMS RIG.
